# Effects of Water Deficit on Growth and Productivity in Tomato Crops Irrigated with Water Treated with Very Low-Frequency Electromagnetic Resonance Fields

**DOI:** 10.3390/plants12213721

**Published:** 2023-10-30

**Authors:** Fernando Ferrari Putti, Jéssica Pigatto de Queiroz Barcelos, Bruno Cesar Goes, Renata Fernandes Alves, Mário Mollo Neto, Alexsandro Oliveira da Silva, Luís Roberto Almeida Gabriel Filho, Willian Aparecido Leotti Zanetti, Angela Vacaro de Souza

**Affiliations:** 1 School of Sciences and Engineering, São Paulo State University (UNESP), Tupã 17602-496, SP, Brazilgabriel.filho@unesp.br (L.R.A.G.F.); angela.souza@unesp.br (A.V.d.S.); 2 School of Agriculture, São Paulo State University (Unesp), Rua José Barbosa de Barros 1870, Botucatu 18610-307, SP, Brazil; jessica.barcelos@unesp.br; 3 Department of Agronomy, José do Rosário Vellano University (Unifenas), Alfenas 37132-440, MG, Brazil; 4 Center of Agrarian Sciences, Federal University of Ceará (UFC), Fortaleza 60356-001, CE, Brazil

**Keywords:** drought stress, EROs, *Solanum lycopersicum* L.

## Abstract

The tomato crop is very sensitive to stress conditions. A water deficit is defined as when precipitation is less than the evapotranspiration (ETc) of the crop in a given period, and in this scenario of climate change, it is identified as responsible for global productivity losses. The use of potential technologies for better irrigation management, such as electromagnetically treated water, remains controversial. Thus, the objective of the present work was to investigate the effects of very low-frequency electromagnetic resonance field treatment on water for tomato crops submitted to different irrigation rates. For this, an experiment was carried out under controlled conditions with different types of water: electromagnetically treated water (WTVLF) and untreated water (UNW), as well as four water replacement rates: 40, 60, 80, and 100% ETc. The electromagnetic treatment of the water was carried out using the commercial equipment AQUA4D^®^. The experiment was carried out in pots with five replications per treatment. Lower activity of SOD, POD, CAT, and APX enzymes was observed in plants irrigated with water treated with very low-frequency electromagnetic resonance fields (WTVLF), indicating less oxidative stress caused by water deficit. Water deficit reduced chlorophyll content, but the effects were less harmful with WTVLF water. The water deficit resulted in less accumulation of dry matter and less productivity in a linear relationship. However, plants irrigated with WTVLF showed increments of about 20% in dry matter accumulation and 20% in fruit production concerning plants irrigated with untreated water, independent of the irrigation rates. We can conclude that irrigation with WTVLF can be a solution to reduce the damage caused by water deficits and increase the productivity of tomato crops.

## 1. Introduction

The tomato (*Solanum lycopersicum* L.) is one of the most produced and consumed vegetables worldwide [1,2]. Its fruit has high nutritional value and is a source of vitamins A and C and carotenoids such as lycopene, which is responsible for the red color of the vegetable [3,4]. Although it can be grown in tropical and subtropical regions [5], the tomato crop is vulnerable to biotic and abiotic stresses [5,6]. Among the environmental factors that most affect tomato growth and productivity, stress due to lack of water (water deficit) stands out [5,6,7,8].

Stomatal closure is the first plant response to water deficit to reduce water loss through transpiration [9]. However, stomatal closure results in the accumulation of electrons derived from the photosynthetic process, increasing the production of reactive oxygen species (ROS) [10]. Thus, from a biochemical point of view, the response of plants to a water deficit is the production of enzymes in the antioxidant system, such as superoxide dismutase (SOD), catalase (CAT), ascorbate peroxidase (APX), and peroxidase (POD), to combat ROS and avoid the cell damage resulting from its accumulation [11,12]. In addition, water deficiency should lead to a reduction in chlorophyll levels [13], inhibition of photosynthesis [14], reduction in leaf area and dry matter production of plants, and, consequently, a reduction in tomato productivity [15].

Irrigation studies on tomatoes to reduce the effects of water stress have become frequent due to extreme conditions resulting from climate change [16,17]. In this sense, proper irrigation management is essential given the need to use water efficiently, directly influencing the productive potential of the crop. More efficient irrigation practices can reduce water application by up to 70% and increase crop productivity by up to 90% [18]. Although some studies have shown that irrigation increases tomato productivity, there is a point, or a specific level, at which productivity stabilizes, no longer responding to irrigation rates [19,20].

In this scenario, eco-friendly technologies emerge to investigate ways to efficiently optimize the use of water in agriculture, seeking agricultural and environmental sustainability [21]. Magnetically treated water is considered a technology of great potential, which concerns a process where water passes through a magnetic field, causing changes in its physical and chemical parameters [7]. Studies show that electromagnetically treated water is an interesting alternative, ranging from seed treatment to the growth and development of seedlings, evidencing changes in the properties of magnetized water in the face of increased hydration of ions, promoting an increase in nutrient and water use rates by plants, increasing their productivity and yield [7,22].

Several studies have reported the effects of water treatment with electromagnetic and magnetic fields. Adopting the same equipment used in this research (AQUA4D^®^), one study found that the treatment effect persists for up to 15 h after the electromagnetic field induction in the water [23].

Such changes are related to the dipole moment of water molecules and the oxygen content [22,24,25], in which they form a small complex of water molecules (e.g., with five or six molecules), which leads to improvements in water activity, such as solubility and reaction rate. Ref. [26] propose that, under a magnetic field, polarized water molecules organize in one direction as hydrogen ions decrease, leading to an increase in pH. Ref. [27] presented a clear explanation of the action of water in the field based on the changes and structure of water through intra-cluster and inter-cluster hydrogen bonds. Magnetization and oxidation treatments alter the physicochemical properties of irrigation water [28], including an increase in pH, electrical conductivity (EC), diffusion, and permeability, and a decrease in viscosity and surface tension when compared to untreated water [29,30]. There are also reports in the literature of changes in viscosity (kg·m^−1^·s^−1^) and surface tension (N/m) that decrease, which facilitates fluidity and permeability [31].

Several studies aim to understand the mechanisms of using water treated with magnetic fields [27,32]. For Populus [33], eggplant [34], wheat [35], and lettuce crops [36], it was found that it provided a reduction in the effects caused by water stress conditions. In addition, studies with germination in tomatoes [37,38] and wheat [39] in saline conditions showed greater development when irrigated with treated water.

Thus, the use of water treated with an electromagnetic or magnetic field is still scarce [40]. In recent years, the use of “eco-friendly” technologies has made researchers seek to understand the mechanism of how treated water behaves in the soil–water–plant relationship. Studies using treated water that aim to reduce the negative effects of water stress have been potentially promising. In barley cultures the characteristic phenological parameters, possible cell damage, electron transport rate, chlorophyll fluorescence, and content of macroelements (Ca, Mg, P, and K) are gradually reduced with increasing MF forces; in opposition, the microelement content (Fe, B, Cu, Mn, Zn, and Mo) is increased in roots [41]. As for cotton crops, there is an increase in the net photosynthetic rate (Pn), transpiration rate (Tr), and instantaneous water use efficiency (iWUE) indexes, and the morphological indexes of cotton seedlings are greater with magnetized fresh water [42], unlike non-magnetized water. Magnetized water irrigation also promotes the growth of rice seedlings and roots [43].

In tomato crops, it has been observed that the use of electromagnetically treated water increases the absorption of nutrients and reduces the occurrence of fruits with symptoms of blossom-end rot [7]. Therefore, we hypothesized that the irrigation of tomato plants with electromagnetically treated water (WTVLF) would reduce the effects of water stress and increase productivity compared to untreated water plants (UNW). Thus, the objective of the present work was to investigate the effect of a very low-frequency electromagnetic resonance field treatment on water for tomato crops, submitted to different irrigation rates.

## 2. Results

The probability values associated with the factors studied are shown in Table 1. There was a significant effect for the type of water applied, in which the content of peroxide (H_2_O_2_) and total chlorophyll b and total dry matter and production showed higher values when irrigated with WTVLF (*p* < 0.05). For the other evaluated components, irrigation with UNW presented higher values (*p* < 0.05). Also, we can infer that there was a significant effect for all components due to the application of different irrigation rates.

### 2.1. Treatment and Experimental Design

The level of H_2_O_2_ and the level of lipid peroxidation, evaluated by the concentration of malondialdehyde (MDA), showed an interaction between the factors (Table 1).

**Figure 1:**
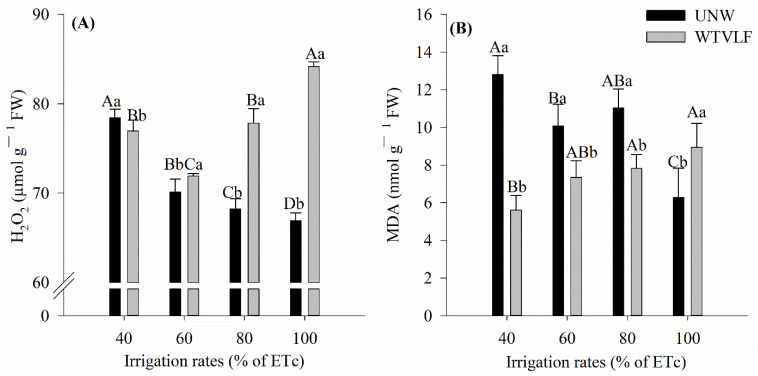
Hydrogen peroxide (H_2_O_2_) (**A**); Lipid peroxidation (MDA) (**B**); in tomato leaf grown at different irrigation rates with two types of water. Means followed by the same letter, lowercase, compare the types of water treatment and, uppercase, compare the different irrigation rates (*p* < 0.05) according to Tukey’s test. Error bars indicate the standard deviation of the mean of five replicates.

The content of H_2_O_2_ as a function of the water type differed in all applied irrigation rates (*p* < 0.05) (Figure 1A). The 100% ETc rate had the greatest difference with an increment of 30% when irrigated with water treated with very low-frequency electromagnetic resonance fields (WTVLF). Except for an ID of 40% ETc, irrigation with UNW led to a lower content of H_2_O_2_. We verified that there was an inverse effect, in which an increase in the irrigation rate of UNW led to a reduction in the content of H_2_O_2_, and, for WTVLF, there was an increase (*p* < 0.05).

There was a reduction in MDA concentration with increasing ID for UNW application (Figure 1B). On the other hand, for the application of WTVLF, the lowest concentration of MDA was obtained with the ID of 40% ETc. Furthermore, except the 100% ETc rate, the application of the other IDs with UNW resulted in higher concentrations of MDA than for WTVLF.

**Figure 2:**
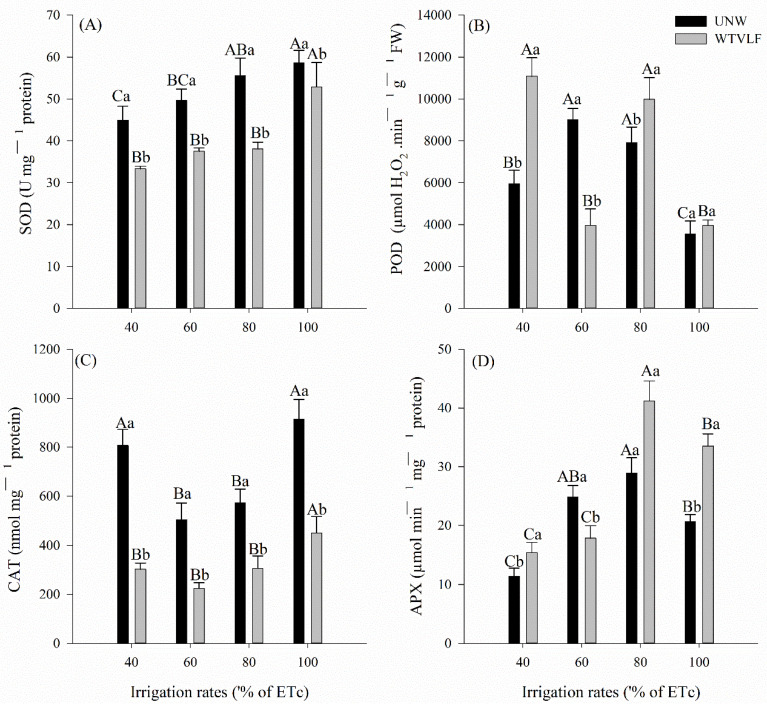
The activity of antioxidant enzymes in tomato crops grown under different irrigation rates with two types of water, representing (**A**) superoxide dismutase (SOD), (**B**) peroxidase (POD), (**C**) catalase (CAT), and (**D**) ascorbate peroxidase (APX). Means followed by the same letter, lowercase, compare the types of water treatment and, uppercase, compare the different irrigation rates (*p* < 0.05) according to the Tukey test. Error bars indicate the standard deviation of the mean of five replicates.

The activity of the SOD enzyme showed an interaction between the factors (Figure 2A) (*p* < 0.05). Irrigation with UNW caused the SOD activity to be higher when compared to the WTVLF for all slides. When observing the effect of water as a function of ID, we observed that there was an increase when irrigated with UNW. As for WTVLF, no differences were observed between treatments irrigated with 40, 60, and 80% ETc. However, significant differences were observed for the 100% ETc rate (*p* < 0.05).

For the POD activity (Figure 2B), there was an antagonistic effect of the irrigation rates, in which there was greater activity at the rates of 40 and 80% ETc when irrigated with UNW and at the rate of 60% ETc when irrigated with WTVLF. For 100% ETc, there was no difference (*p* > 0.05). Nevertheless, for irrigation with UNW, the 40 and 80% ETc irrigation rates showed higher POD enzyme activity than the 60 and 100% ETc rates. In addition, the use of WTVLF presented superior activity for 60 and 80% ETc, compared to the other rates.

The highest activity of the catalase enzyme occurred when irrigated with UNW for all slides (Figure 2C). When analyzing the effect of the rate for UNW, there was a difference between the rates of 40 and 100% ETc compared to that of 60 and 80% ETc (*p* < 0.05). When irrigated with WTVLF, the rate of 100% ETc differed from the others (*p* < 0.05). The activity of the enzyme APX (Figure 2D) had an effect due to the type of water and irrigation rate, in which, at 60% ETc, there was a greater activity for irrigation with UNW. The others had a positive effect when irrigated with WTVLF. The highest activity for both types of water was found at 80% ETc.

### 2.2. Effects of Water Stress on Chlorophyll and Tomato Production

**Figure 3:**
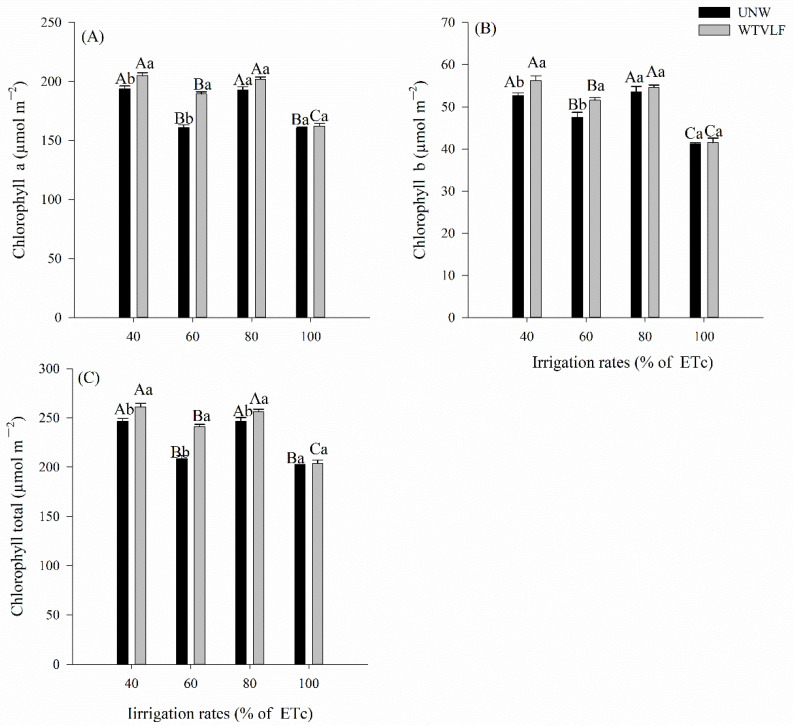
The chlorophyll of tomatoes cultivated in different irrigation rates with two types of water. (**A**) Chlorophyll a, (**B**) chlorophyll b, and (**C**) total chlorophyll. Means followed by the same letter, lowercase, compare the types of water treatment and, uppercase, compare the different irrigation rates (*p* < 0.05) according to the Tukey test. Error bars indicate the standard deviation of the mean of five replicates.

The interaction between the water type and irrigation rate significantly affected the content of chlorophyll a, b, and total (Figure 3). We can highlight that the behaviors of the 40 and 60% ETc levels were similar for chlorophyll a and b, in which the irrigation with WTVLF presented higher levels (*p* < 0.05). However, for the application of IDs of 80 and 100% ETc, the water types did not differ among themselves in terms of chlorophyll a and b (Figure 3A,B). The total chlorophyll content in the leaves showed an effect of the adopted water type, in which only the 100% ETc rate showed no difference (*p* > 0.05). Furthermore, analyzing the impact of ID, the concentrations of chlorophylls a, b, and total (Figure 3C) were higher in the function of the irrigation rates of 40 and 80% ETc.

**Figure 4:**
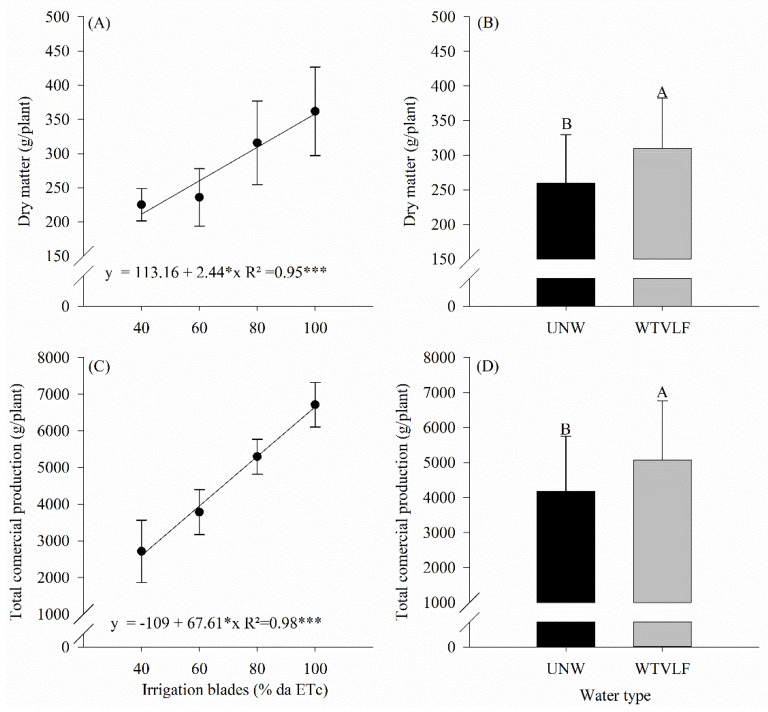
Total dry matter accumulation (**A**,**B**) and total commercial production (**C**,**D**) of tomato cultivated under different irrigation rates and two water types. Means followed by the same letter, lowercase, compare the types of water treatment and, uppercase, compare the different irrigation rates (*p* < 0.05) according to Tukey’s test. Error bars indicate the standard deviation of the mean of five replicates. * multiplicate and *** significative a 1%.

The dry matter accumulation showed no interaction between the factors (Figure 4A,B). Thus, increasing irrigation rates resulted in a linear increase in plant dry matter production, with each 1% increase in ETc causing an increase of 2.44 g plant^−1^ (Figure 4A). As for the applied water type, irrigation with WTVLF showed a 20% increase in dry matter accumulation (Figure 4B).

As with dry matter accumulation, commercial production showed no interaction between factors (Figure 4C,D). There was a linear effect of the irrigation rate, in which a 1% increase in ETc caused an increase in the production of 67.61 g plant^−1^ (Figure 4C). Regarding the applied water type, irrigation with WTVLF provided an average increase of 20% in production per plant (Figure 4D).

**Figure 5:**
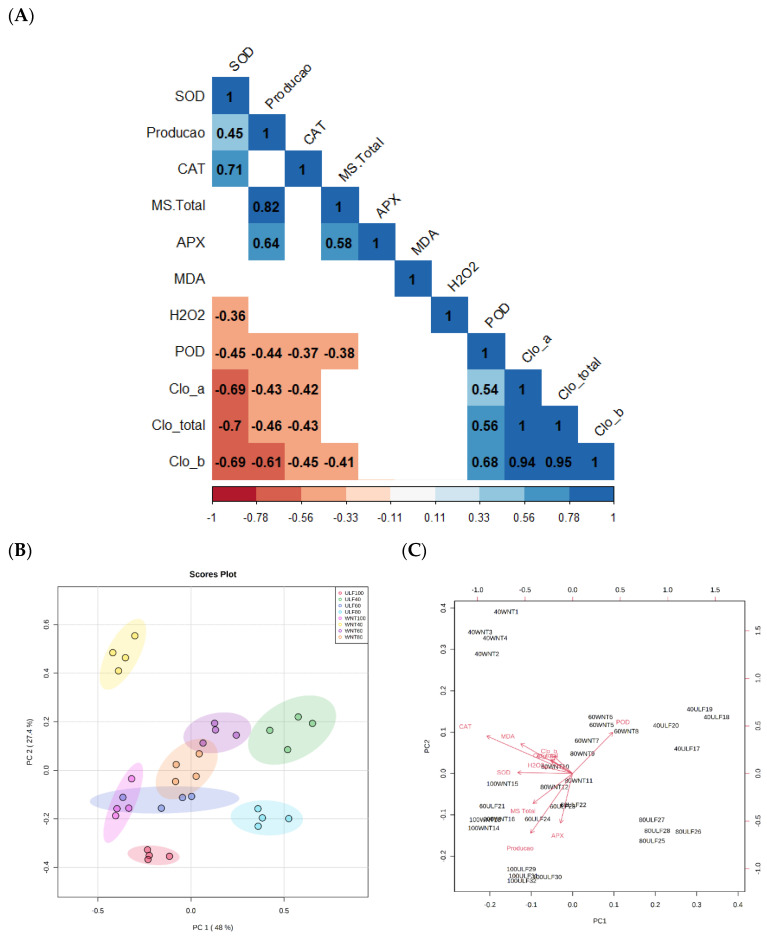
Heatmap for Pearson’s correlation coefficients (**A**), and principal component analysis (PCA) (**B**,**C**) for tomato cultivation submitted to different irrigation rates and water types. The heatmap graph shows only significant values (*p* < 0.05).

Figure 5A presents a heatmap of the correlations found between the variables analyzed in the experiment. We can thus observe a strong correlation (above 0.94) between the chlorophyll contents. Another important result regards the positive correlations between production and dry matter (DM), total, and APX activity and the negative correlation between production and DM, total, POD, and chlorophyll contents. In addition, the activity of the SOD enzyme correlated positively with production and the activity of the CAT enzyme and negatively with H_2_O_2_, POD, and chlorophyll content (Figure 5A).

Figure 5B,C present the PCA and its components, respectively. We can observe that the irrigation rates of 40, 60, and 80% ETc irrigated with UNW had a greater influence on components of the activity of CAT, MDA, and SOD. It also had a lower weight of components related to chlorophyll contents. The component of POD activity was associated with the treatment of 40% ETc irrigated with WTVLF. In addition, the 100% ETc treatments with WTVLF and UNW were strongly influenced by the production component and total dry matter.

## 3. Discussion

### 3.1. Effects on the Tomato Antioxidative System

The tomato crop is classified as sensitive to water deficits. The first changes promoted by a water deficit that can be noted happen in plant morphology, physiology, and biochemistry, thus leading to various disorders [44]. These changes are reported as reduced photosynthetic activity [45,46], biochemical changes [47,48], and, finally, impact on production [49].

The oxidative system is directly or indirectly altered in the plant, in which cell membrane damage occurs, totally altering their functioning and leading to metabolic disorders [50,51]. The ROS produced must be eliminated as quickly as possible, reducing electrolyte leakage and lipid peroxidation to maintain the integrity of organelles and cell membranes [52].

Water stress induces antioxidant stress, generating ROS, which can produce O_2_^•−^, hydroxyl radicals (OH•), singlet oxygen (_1_O^2^), and H_2_O_2_ [53]. The accumulation of ROS causes the degradation of lipids, with the formation of malondialdehyde (MDA) as the end product of cell wall degradation [52,54,55]. Numerous studies conducted under water deficit conditions have found enhanced activities of essential antioxidant enzymes such as SOD, POD, CAT, and APX to control the production of O_2_^•−^ and H_2_O_2_ in tomato crops [13,54,56,57]. According to [58], APX uses ascorbate as a substrate to stimulate the conversion of H_2_O_2_ to H_2_O, and its activity is generally elevated under stress conditions. This effect was observed at work with greater intensity for the 80 and 100% ETc rates with the application of WTVLF (Figure 2D). Yet, APX activity proved to be sufficient to control ROS levels, as the MDA concentration for these treatments was not the highest observed (Figure 2) [59].

Thus, plants irrigated with WTVLF would have greater ease at absorbing the available water under lower irrigation rates, providing less oxidative stress compared to plants irrigated with untreated water. This result is evident when we compare the MDA concentrations between treatments, which remained at lower levels for plants treated with WTVLF. As mentioned earlier, MDA is related to cell membrane damage and, therefore, displays an insufficient performance of antioxidant enzymes in the face of stress [52,54,55]. In an experiment with water stress in tomatoes, ref. [60] found high concentrations of MDA where water stress was severe. Increases in MDA concentration were also observed by [54] for irrigation rates of 60% ETc.

### 3.2. Effects of Water Stress on Chlorophyll and Production

Water stress affects chlorophyll contents, which are extremely important for photosynthesis. One of the main causes for the decline in the amount of chlorophyll due to water stress is the O_2_^•−^ and H_2_O_2_ promoted by drought, which results in lipid peroxidation and, finally, chlorophyll degradation [61]. Reduction of chlorophyll content has also been observed in plants under water stress and at high temperatures [54].

The mode of action of water induced by a magnetic or electromagnetic field is partially broken by its hydrogen bonds. In addition, some water molecules become free monomer molecules that can more easily penetrate the walls of biological cells, thus promoting plant growth [30]. Ref. [62] stated that the increase in cell division and enlargement can be attributed to an increase in the activities of the enzymes gibberellic acid (GA3) and indole acetic acid (IAA), cytokinin synthesis, and reduction of abscisic acid (ABA).

The increase in chlorophyll content agrees with that in cowpea plants, in which it has been found that treating plants with magnetic technologies caused a significant increase in photosynthetic pigment concentrations. Higher levels of photosynthetic pigments, when using magnetically treated water, indicate the effect of activating magnetic fields on the concentration of ions such as K^+^ and GA_3_ [35], which leads to an increase in the number of chloroplasts per cell. Ref. [63] suggested that magnetic technology increased the phospholipid/sterol ratio, causing an increase in the fluidity of the membrane lipid bilayer, while sterols act as a barrier to prevent leakage in biological membranes.

The increase in growth and yield of wheat plants irrigated with magnetic water may be due to changes in the transport of assimilates, enzymatic activity, growth regulators, ions and water uptake [64], and/or an energetic excitation of one or more cellular substrate parameters, such as proteins and carbohydrates [65]. In tomato plants subjected to saline stress, irrigation with WTVLF provided higher plant growth and higher fruit yield [7]. The authors also observed a better nutritional status of the plants, as well as a lower occurrence of blossom-end rot symptoms in the fruits [7].

Regarding understanding the behavior of WTVLF, there are still some gaps. Nevertheless, we know that water is more available in the soil, that is, studies indicate that there is a direct influence on soil tension and moisture [6,66,67,68,69].

## 4. Materials and Methods

### 4.1. Experimental Area Characterization

The experiment was carried out at the Experimental Farm of the Faculty of Sciences and Engineering, Tupã city campus, São Paulo State, Brazil, whose geographic location is defined by the coordinates 22° 51′ south latitude (S) and 48° 26′ west longitude (W) and an average altitude of 786 m above sea level. The monthly average wind speed at 10 m height is 3.1 ms^−1^ and the daily monthly average global solar energy is 4772.13 Wh m^−2^. The greenhouse was 6 m wide, 27 m long, and 4 m tall, and was coated with transparent polyethylene plastic film with an anti-UV additive on top (150 μm thick) as well as aphid screens on the sides.

The climate is characterized as AW, according to the Koppen classification, meaning hot and humid with summer rains, with an annual mean air temperature of 27.1 °C and an annual precipitation mean of 2423 mm. The experiment was conducted between 3 October and 20 December 2021.

Temperature and humidity data were collected daily at 9 a.m., recorded by a datalogger located at the center of the greenhouse and approximately 2 m high. A Class A tank was also installed at the center of the greenhouse to collect evaporation data.

The soil used in the experiment is classified as eutrophic red–yellow argisol [70], with a sandy/medium texture and electric conductivity around 0.50 d Sm^−1^. Table 2 presents the results of the physical and chemical analyses of the soil sampled at a rate between 0 and 0.20 m (Table 2).

Before installing the experiment, the necessary corrections were carried out in the soil to raise the base saturation to 80%, applying dolomitic limestone (relative neutralizing value, RNV = 91%), according to recommendations [71].

Fertilization was performed according to [72], in which the application recommendations before planting were 70 kg ha^−1^ of N; 600 kg ha^−1^ of P_2_O_5_; 200 kg ha^−1^ of K_2_O; 30 kg ha^−1^ of sulfur; 2 kg ha^−1^ of boron; and 4 kg ha^−1^ of zinc. Cover was applied in 8 installments at 10-day intervals, with the recommendation of 300 kg ha^−1^ of N; 150 kg ha^−1^ of P_2_O_5_; and 300 kg ha^−1^ of K_2_O divided over each installment.

The seedlings were sown in polyethylene trays with a capacity of 50 seeds and filled with substrate. Thirty-five days after sowing, the seedlings were transplanted into polyethylene pots with a volumetric capacity of 12 dm^−3^. Following a recommendation [73] for the tomato crop, we adopted a spacing between lines of 1 m in width and 0.25 m between plants. The pots were placed on an iron structure, so that they were 0.20 m from the ground, avoiding contact with it. The plants were tutored using ribbons tied to the plant stem and to the aluminum structure in the greenhouse.

Irrigation at 100% ETc was kept constant for the first 20 days to stabilize the culture. Afterward, the irrigation was differentiated according to the treatments.

The air temperature measured inside the greenhouse during the experiment had a maximum of 36.8 °C and an average of 19.39 °C, with a variation of 17.41 °C. The indoor air humidity determined had a maximum of 98.4% and an average of 40.9%, with a variation of 57.5 °C. The total Eca accumulated during the period was 311 mm, with an average of 3.11 mm day^−1^.

### 4.2. Treatment and Experimental Design

The experiment was carried out in a 2 × 4 factorial scheme with 5 replications, as follows: (i) two types of irrigation water (untreated water, UNW, and water treated with very low-frequency electromagnetic resonance fields, WTVLF); and (ii) four irrigation rates (40, 60, 80, and 100% of tomato ETc). The water treatment was performed by commercial AQUA4D^®^ equipment, as described in the following section.

For proper irrigation management, the reference evapotranspiration was estimated by a Class A tank. Two independent drip irrigation systems were used, one with water treated with very low-frequency electromagnetic resonance fields (WTVLF) and another with untreated water (UNW).

Irrigations were carried out by drip using emitters with a nominal flow of 2 L h^−1^. To estimate the reference evapotranspiration (ETo), an evaporimeter tank was used, installed inside the greenhouse, according to [73,74,75], in which measurements were performed daily. ETo was determined using Equation (1), defined by [76]:

ETo = EV × Kt,(1)

where ETo is the reference evapotranspiration (mm d^−1^); Kt is the tank coefficient (dimensionless); and EV is the tank evaporation (mm d^−1^).

The correction of the tank coefficient was estimated by [76], according to the following equation:

Kp = 0.482 + 0.025(ln(b) − 0.00376 × S + 0.0045 × UR)(2)

where B is the distance around the Class A tank of vegetation; S is the wind speed at 2 m altitude (km day-); and UR is the relative humidity (%).

We emphasize that the values of B and S were disregarded, as the experiment was conducted in a greenhouse.

The gross rate applied was calculated using Equation (3):

LB = (Eto × Kc)/Ef,(3)

where LB is the raw water rate (mm d^−1^); Eto is the reference evapotranspiration (mm d^−1^); Kc is the crop coefficient (dimensionless); and Ef is the irrigation system efficiency (0.90).

The crop coefficient (Kc) used in each tomato development stage was adapted from the recommendation [77]. Stage I: from transplanting to 10% of vegetative development (0.60); Stage II: from the end of Stage I to the beginning of the flowering phase (0.85); Stage III: from the end of Stage II to the beginning of maturation (1.15); and Stage IV: from the end of Stage III to the end of harvest (0.90).

Irrigation intensity was determined using Equation (4):

Ia = (n × v)/ec,(4)

where Ia is the application intensity (mm h^−1^); n is the number of emitters per plant; v is the emitter flow (L h^−1^); and ec is the area of the vessel (m^2^).

The irrigation time was calculated according to Equation (5):

Ti = LB/Ia,(5)

where Ti is the irrigation time (h); LB is the raw rate (mm d^−1^); and Ia is the application intensity (mm h^−1^).

The equipment used for the water electromagnetic treatment was the AQUA4D^®^, which performs a physical treatment in the water. Quantum physics indicates that water is organized and structured matter, and not chaotic, as one might think. Water is an element that can adapt to very different structures. Thus, when water passes through an electromagnetic field, it provides a better dissolution and distribution of minerals in the water thus causing an increase in water retention in the soil as well as a better adsorption of minerals by plants. As such, the equipment consists of an electronic panel pre-programmed to generate electromagnetic signals and a tube that the water passes through, where it receives the aforementioned signals.

The water after treatment has effects for up to 15 h of magnetic field tracking. The magnetic field fluctuation recorded is relatively low with Bmax = 0.023 mT and Bmin = 0.008 mT, with Bavg = 0.0155 mT for magnetic water, while for tap water, the estimated values were Bmax = 0.0185 mT and Bmin = 0.005 mT, with Bavg = 0.01175 mT [23].

To carry out the biochemical analyses of this project, leaf samples were collected from four tomato plants per treatment. After collection, the leaves were placed in 45 mL Falcon tubes, and liquid nitrogen was immediately added for rapid freezing of the samples. Then, the tubes were kept in a freezer at −80 °C. To process the leaves, macerations were carried out in a mortar with liquid nitrogen until fine powder particles were obtained, which were properly weighed and transferred to tubes, and stored again in a freezer at −80 °C [78]. The last total expanded leaves were collected for enzymatic extraction. After collection, they were stored in liquid N until processed. For homogenization, a ball mill was used, in which the samples were macerated.

### 4.3. Determination of Enzyme Activity

The processed extract was used to analyze the protein concentration and enzymatic activities (superoxide dismutase, SOD; and catalase, CAT) of the tomato samples. The plant material was obtained by resuspension of the processed extract, using a mass of 200 mg in 4.0 mL of 0.1 M potassium phosphate buffer, at pH 7.8, and adding 300 mg of PVPP (polyvinylpolypyrrolidone). The mixture was centrifuged for 20 min at 5000× *g*, and the supernatant was separated, collected in tubes, and stored in a freezer at −80 °C. The concentration of soluble protein present in the samples was determined according to the method in [79], well established in the literature for this purpose. Finally, bovine serum albumin (BSA) was used as a standard.

The activity of the enzyme superoxide dismutase (SOD—EC 1.15.1.1) was determined according to the protocol described by [80,81]. The determination of catalase (CAT—EC 1.11.1.6) was performed by the method of [82]. Ascorbate peroxidase activity (APX—EC 1.11.1.11) was determined according to the methodology of [83]. The determination of POD activity was performed using the technique described by [84].

### 4.4. Determination of the Total Soluble Protein Content

To analyze the content of hydrogen peroxide (H_2_O_2_), the methodology described by [85] was used. For the determination of malondialdehyde (MDA), the methodology described by [86] was used.

### 4.5. Determination of Chlorophylls

The determination of chlorophylls a, b, and total was performed using the technique described by [87].

### 4.6. Determination of Production and Dry Mass

The determination of the dry mass of the aerial part was carried out at the end of the experiment. The plants were cut close to the ground, packed in a paper bag, and taken to a dry air oven at 65 °C for 4 days. Afterward, they were weighed and thus the accumulation of dry matter in the plant was determined.

### 4.7. Analyses Statistics

Data normality was verified using the Kolmogorov—Smirnov statistical test. For data with a normal distribution, analysis of variance (ANOVA) was performed with subsequent use of Tukey’s test. In this case, for the analyses that present interaction between the factors (types of water and levels of conductivity), the averages were compared by first contrasting the levels of one of the factors with each of the levels of the other factor, and vice-versa. When there was no interaction between the factors, comparisons were made between the means only of the factor levels that showed a significant difference in ANOVA. A confidence level of 5% was considered in all statistical tests [88] and the R software [89] was used for the application of the described tests.

Pearson’s correlation analysis (*p* < 0.05) was performed using the R software (R Development Core Team). For the elaboration of the heatmap, the packages ‘corrplot’, ‘latticeExtra’, and ‘RColorBrewer’ were used. Principal component analysis was also performed in the R software, in which the ‘PCAtools’, ‘chemometrics’, and ‘stats’ packages were used. The ‘ellipse’ package was used for the construction of the figures. For the construction of the heatmap and hierarchical clustering, the Euclidean distance algorithm was used [90,91,92].

## 5. Conclusions

Water deficits are a reality to which producers are increasingly susceptible, due to climate change and its consequent shift in rainfall regimes. As such, technology and management that seek to reduce the impacts caused by these changes are of fundamental importance and need to be elucidated.

An increase in the supply of irrigation suitable for tomato crops provides less oxidative stress, greater accumulation of dry matter, and greater production of commercial fruits. However, it was possible to verify a reduced impact of water deficit on oxidative stress in plants irrigated with electromagnetically treated water (WTVLF). In addition, the use of WTVLF resulted in a greater accumulation of dry matter and greater production of commercial fruits, regardless of the used irrigation rate. In this way, we conclude that the use of the technology studied in this article can be an alternative to help reduce the impacts of the water deficit and also a way to increase production.

## Figures and Tables

**Table 1:** Probability (p) of the values associated with the factors: water type (WT), irrigation rates (ID), the content of peroxide (H_2_O_2_) and foliar malondialdehyde (MDA), the activity of antioxidant enzymes (CAT, APX, SOD, POD), chlorophylls (Cl a/b/total), and biometric components, total dry matter (DM), and production.

Variation Sources	H_2_O_2_ (µmol g ^−1^ FW)	MDA (nmol g^−1^ FW)	CAT (nmol mg^−1^ protein)	APX (µmol mg^−1^ protein)	SOD (U mg^−1^ protein)	POD (µmol H_2_O_2_ min^−1^ g^−1^ FW)	Chl a (µmol m^−2^)	Chl b (µmol m^−2^)	Total Chl (µmol m^−2^)	D.M. (g)	Production (kg)
WT	<0.001	<0.001	<0.001	<0.001	<0.001	0.018	<0.001	<0.001	<0.001	0.006	0.003
ID	<0.001	0.01	<0.001	<0.001	<0.001	<0.001	<0.001	<0.001	<0.001	<0.001	<0.001
WT × ID	<0.001	<0.001	0.003	<0.001	0.012	<0.001	<0001	0.005	<0.001	0.1	0.9
VC	0.68	12.32	11.32	8.94	6.96	9.50	2.69	1.92	12.41	13.80	10.20
WT UNW	70.93 ^b^	10.06 ^a^	699.33 ^a^	21.46 ^b^	52.16 ^a^	7249.99 ^a^	177.0 ^b^	48.74	225.76 ^b^	259.33 ^b^	4176.66 ^b^
WTVLF	77.85 ^a^	7.43 ^b^	320.55 ^b^	27.00 ^a^	40.44 ^b^	6605.82 ^b^	189.7 ^b^	50.98 ^a^	240.69 ^a^	309.90 ^a^	5071.66 ^a^
ID (%) 40	78.21	9.21	554.71	13.44	39.11	8517.60	199.5	54.46	253.91	225.15	2710
60	70.65	8.71	363.77	21.34	43.59	6481.17	175.2	49.55	224.77	235.85	3780
80	73.04	9.43	439.28	35.07	46.79	8950.76	197.4	54.06	251.46	315.63	5290
100	75.67	7.62	682.01	27.08	55.71	3762.13	16.1.4	41.36	202.76	361.85	6713

Description: VC: Variation coefficient; DM: dry matter; UNW: untreated water; WTVLF: electromagnetically treated water. Means followed by the same letter, lowercase, compare the types of water treatment (*p* < 0.05) according to Tukey’s test.

**Table 2:** Chemical and physical characteristics of the soil.

Chemical Characteristics									
Al^3+^	H + Al	Na	K	Ca	Mg	SB	CTC	V%	
mmolc/dm^3^									
0	11	-	2.7	8	4	15	26	57	
pH	pH	M.O.	Presin	S	B	Cu	Fe	Mn	Zn
CaCl_2_	H_2_O	g/dm^3^	mg/dm^3^	mg/dm^3^					
5.1	-	6	3	4	0.14	0.5	18	28.4	0.8
Physical characteristics									
	Sand			Clay			Silt		
(g.kg^−1^)									
	915			38			47		
